# Author Correction: Overshooting the critical threshold for the Greenland ice sheet

**DOI:** 10.1038/s41586-023-06852-5

**Published:** 2023-11-13

**Authors:** Nils Bochow, Anna Poltronieri, Alexander Robinson, Marisa Montoya, Martin Rypdal, Niklas Boers

**Affiliations:** 1 Department of Mathematics and Statistics, UiT – The Arctic University of Norway, Tromsø, Norway; 2 Physics of Ice, Climate and Earth, Niels Bohr Institute, University of Copenhagen, Copenhagen, Denmark; 3 Potsdam Institute for Climate Impact Research, Potsdam, Germany; 4 Alfred-Wegener-Institut, Helmholtz-Zentrum für Polar- und Meeresforschung, Potsdam, Germany; 5 Department of Earth Science and Astrophysics, Complutense University of Madrid, Madrid, Spain; 6 Instituto de Geociencias, CSIC-UCM, Madrid, Spain; 7 Earth System Modelling, School of Engineering & Design, Technical University of Munich, Munich, Germany; 8 Department of Mathematics and Global Systems Institute, University of Exeter, Exeter, UK

**Keywords:** Projection and prediction, Cryospheric science, Climate-change impacts

Correction to: *Nature* 10.1038/s41586-023-06503-9 Published online 18 October 2023

In the version of the article initially published, we referred to the surface mass balance module of PISM as dEBM. However, the surface mass balance module introduced by Zeitz et al.^1^ and used in PISM is a modified version of the full dEBM scheme introduced by Krebs-Kanzow et al.^2,3^. To make this distinction clearer, we now clearly state that we use dEBM-simple and added an additional explanation in the Methods. We thank Uta Krebs-Kanzow for pointing this out.
